# Monitoring LC3- or GABARAP-positive autophagic membranes using modified RavZ-based probes

**DOI:** 10.1038/s41598-019-53372-2

**Published:** 2019-11-12

**Authors:** Sang-Won Park, Pureum Jeon, Yong-Woo Jun, Ju-Hui Park, Seung-Hwan Lee, Sangkyu Lee, Jin-A. Lee, Deok-Jin Jang

**Affiliations:** 1 Department of Ecological Science, College of Ecology and Environment, Kyungpook National University, 2559, Gyeongsang-daero, Sangju-si, Gyeongsangbuk-do 37224 Republic of Korea; 2 Department of Biological Science and Biotechnology, College of Life Science and Nano Technology, Hannam University, 1646, Yuseong-daero, Yuseong-gu, Daejeon 34054 Republic of Korea; 3 Center for Cognition and Sociality, Institute for Basic Science (IBS), Daejeon, 34126 Republic of Korea

**Keywords:** Autophagy, Macroautophagy

## Abstract

Xenophagy is a selective lysosomal degradation pathway for invading pathogens in host cells. However, invading bacteria also develop survival mechanisms to inhibit host autophagy. RavZ is a protein secreted by *Legionella* that irreversibly delipidates mammalian autophagy-related protein 8 (mATG8) on autophagic membranes in host cells via efficient autophagic membrane targeting. In this study, we leveraged the autophagic membrane-targeting mechanism of RavZ and generated a new autophagosome probe by replacing the catalytic domain of RavZ with GFP. This probe is efficiently localized to mATG8-positive autophagic membranes via a synergistic combination between mATG8 protein-binding mediated by the LC3-interacting region (LIR) motifs and phosphoinositide-3-phosphate (PI3P) binding mediated by the membrane-targeting (MT) domain. Furthermore, the membrane association activity of this new probe with an MT domain was more efficient than that of probes with a hydrophobic domain that were previously used in LIR-based autophagosome sensors. Finally, by substituting the LIR motifs of RavZ with selective LIR motifs from Fyco1 or ULK2, we developed new probes for detecting LC3A/B- or GABARAP subfamily-positive autophagic membranes, respectively. We propose that these new RavZ-based sensors will be useful for monitoring and studying the function of mATG8-positive autophagic membranes in different cellular contexts for autophagy research.

## Introduction

Xenophagy is selective autophagy by which host cells degrade invading pathogens in lysosomes^1,2^. However, many bacteria have developed a survival mechanism to escape host autophagy by inhibiting the functions of host autophagic proteins^1,3,4^. One component that is essential to mammalian autophagy is mATG8, a mammalian homolog of yeast autophagy-related protein 8. mATG8 plays key roles in autophagosome formation, cargo recognition, and the recruitment of cargos into the autophagosomal membrane^5–9^. In mammals, there are two subgroups of ATG8-like proteins: microtubule-associated protein light chain 3 (LC3) proteins LC3A, LC3B, and LC3C and γ-aminobutyric acid receptor-associated proteins (GABARAPs) GABARAP, GABARAP-L1, and GABARAP-L2^10,11^. These proteins are lipidated by phosphatidylethanolamine (PE) conjugation to their C-terminal regions and are incorporated into membranes depending on different cellular contexts, leading to autophagosome formation and maturation^6,11–14^. However, few methods are available to date for monitoring the cellular localization of each endogenous LC3-, GABARAP-subfamily protein in live cells, and changes to cellular localization in certain physiological or pathogenic conditions^15,16^.

RavZ is a cysteine protease that is secreted from the intracellular pathogen *Legionella pneumophila* into the cytoplasm of host cells and irreversibly delipidates mATG8-PE proteins in autophagic membranes by hydrolyzing the amide bond between the C-terminal glycine residue and an adjacent aromatic residue, impairing autophagosome formation and ultimately inhibiting xenophagy in host cells^17^. To efficiently inhibit host autophagy, RavZ must be properly targeted to autophagosomes. RavZ has two LC3-interacting region (LIR) motifs at the N-terminal region (LIR1/2 motifs) and one LIR motif at the C-terminal region (LIR3 motif) that bind to mATG8 proteins in autophagosomes^18,19^. In addition to these LIR motifs, there is a catalytic domain and a phosphatidylinositol 3-phosphate (PI3P)-binding membrane-targeting (MT) domain in RavZ. Since PI3P is enriched in pre-autophagosomal and autophagosomal membranes, a PI3P-binding MT domain might lead to the targeting of RavZ into high-curvature autophagic vesicles^20^.

In addition to our interest in elucidating the functions of RavZ in host cells, we also became interested in understanding the targeting mechanism of RavZ into autophagosomal membrane since our group and others have recently developed LIR-based autophagosome sensors to detect endogenous autophagosomes^15,16,21^. Our group developed autophagosome sensors using LIR motifs and hydrophobic domains (HyD) with enhanced membrane association that efficiently detect endogenous mATG8-positive autophagosomes^11,15^. Other groups have identified selective mATG8-binding peptides by screening peptide libraries using phage display screening. Combining these peptides with the PB1 (Phox/Bem 1p) domain of p62, which helps self-oligomerization, they developed selective autophagosome sensors, including an LC3C-specific probe^16^. HyD and PB1 domains have been used for efficient targeting of LIR-based autophagosome sensors, but their assisting mechanisms are different. A HyD domain assists membrane association of the probe on the autophagosome, while a PB1 domain induces multimerization of LIR motifs, leading to the enhancement of autophagosome targeting via multiple mATG8 associations on the autophagic membrane. However, within the cells, there are many PB1 domain-containing proteins to interfere with the function of the PB1-containing probe, and multimers of LIR motifs also have increased non-specific binding with other proteins, including other LC3- or GABARAP-subfamily proteins. Therefore, using a membrane association domain instead of a dimerization/multimerization domain might have an advantage^22^. PI3P is involved in the formation and the regulation of autophagosome maturation, although it also exists in the endosomal membrane^23–28^. Therefore, PI3P binding motifs are good candidates for assisting the probes in associating with autophagic membranes if combined with an LIR motif. There are several PI3P-binding motifs, including conserved region 2 (C2), Fab1 YOTB Vac1 EEA1 (FYVE), phox homologue (PX), pleckstrin‐homology domain (PH), GRAM-Like Ubiquitin-binding in EAP45 (GLUE) and glucosyltransferase, Rab‐like GTPase activator, and myotubularin (GRAM) domains^29^. Among these PI3P motifs, an FYVE motif was used to enhance autophagosome detection in a previous study, but it was less efficient as a probe than a PB1 domain^16^. However, if strong PI3P binding domains are used for the probes, they are basally localized to early endosomes and sequester and alter PI3P dynamics in cells. Therefore, weak PI3P-binding domains that are not localized to, but help the localization of the proteins into early endosomes or autophagosomes, can minimize inhibition effects on PI3P function and are therefore useful for the generation of autophagosome-detecting probes.

Interestingly, RavZ has a unique PI3P binding MT domain, which helps autophagosome targeting via membrane association^17,20^. Although MT domains and LIR motifs of RavZ can be involved in autophagosome targeting, their contributions remain elusive. Therefore, in this study, we tested the possibility of constructing new autophagosome probes using the PI3P-binding MT domain and LIR motifs from RavZ to enhance its autophagosome targeting. To do this, RavZ(ΔCA)-GFP was generated by replacing the RavZ enzyme activity domain with GFP. RavZ(ΔCA)-GFP was efficiently localized to autophagic membranes through mATG8 binding mediated by LIR motifs and PI3P binding mediated by an MT domain within the RavZ protein. An MT domain or LIR motif alone was insufficient or weak for autophagic membrane targeting. However, an MT domain combined with one or more LIR motifs leads to efficient targeting of the RavZ-based sensor to autophagic membranes. Interestingly, an MT domain was even more efficient than a HyD domain for facilitating autophagic membrane targeting. Furthermore, to increase selective targeting of RavZ-based sensors into LC3- or GABARAP-positive autophagic membranes, we replaced the LIR motifs of RavZ with selective LC3- or GABARAP subfamily-binding LIR motifs and developed additional RavZ-based probes that were selectively detecting for LC3- or GABARAP-positive autophagic membranes in cells. Thus, compared to HyD-LIR(x)-GFP, our newly developed RavZ-based fluorescent autophagosome probes are potentially useful for monitoring mATG8 family proteins in autophagy research with different types of cells under physiological or pathological conditions.

## Results and Discussion

### Generation and cellular localization of RavZ(ΔCA)-GFP into LC3 or GABARAP-positive autophagic membranes in an MT domain, LIR1/2 motif, or LIR3 motif-dependent manner

**Figure 1:**
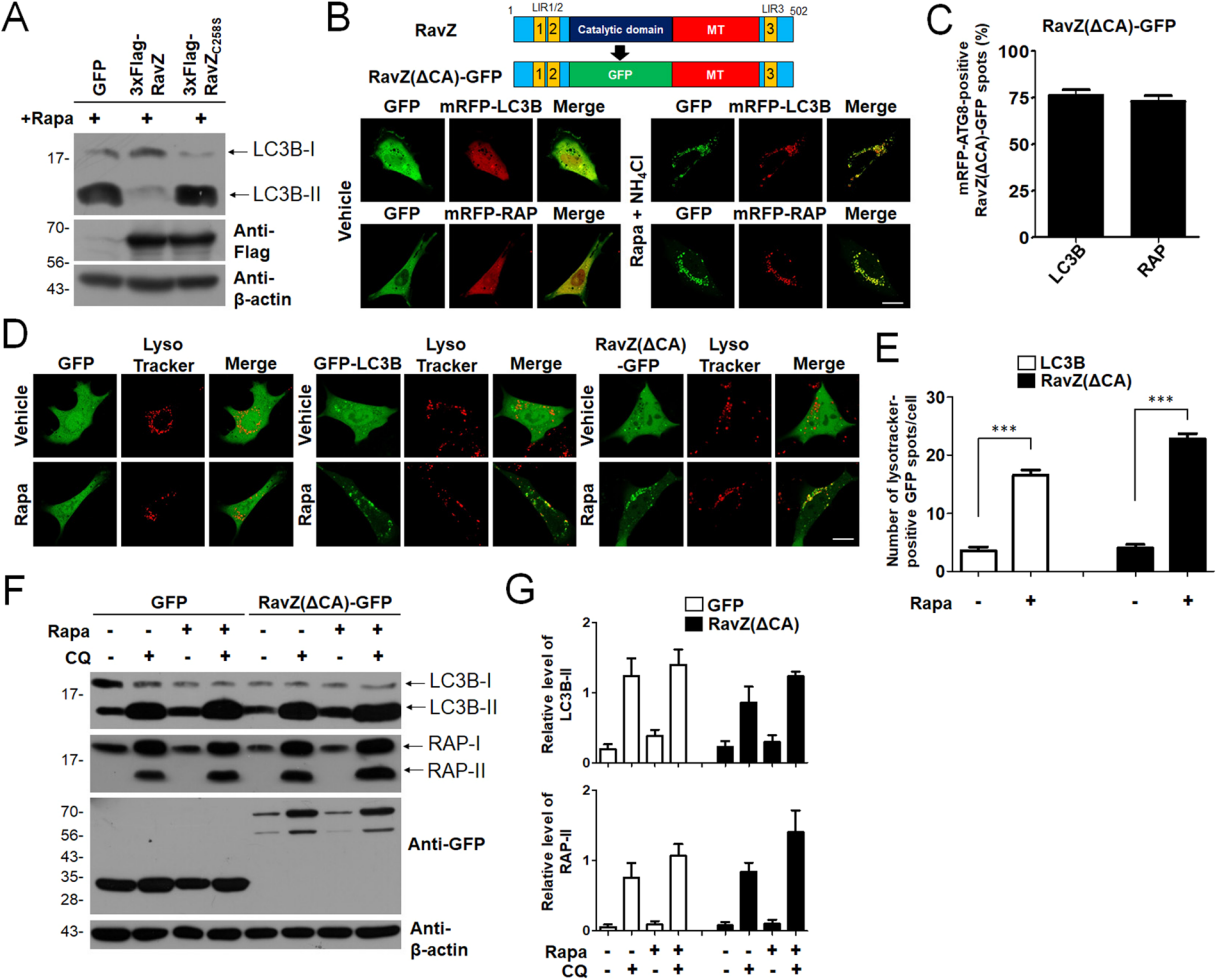
Efficient autophagosome targeting of RavZ(ΔCA)-GFP. (**A**) Representative Western blots of endogenous LC3B in cells expressing 3xFlag-RavZ protein or 3xFlag-RavZ_C258S_ catalytic mutant in HEK293T cells upon autophagy induction (100 nM rapamycin). Extended blot images including these data are presented in Supplementary Fig. 5A. (**B**) Schematic diagram of GFP-fused RavZ mutant protein (RavZ(ΔCA)-GFP) (upper) and confocal images depicting the cellular localization of RavZ(ΔCA)-GFP co-expressed with mRFP-LC3B or mRFP-GABARAP in MEF cells treated with 100 nM rapamycin (rapa) + 10 mM NH_4_Cl. Scale bar, 10 µm. (**C**) The bar graphs illustrate the percentages of mRFP-LC3B- or mRFP-GABARAP-positive RavZ(ΔCA)-GFP spots (*n* = 25 for each group). (**D**,**E**) Confocal images showing cellular localization of GFP, GFP-LC3B or RavZ(ΔCA)-GFP with Lysotracker into MEFs upon 100 nM rapa treatment. The bar graph illustrates the ratios of LysoTracker-positive RavZ(ΔCA)-GFP spots number per cell (*n* = 25 for each group). The data are presented as the mean ± SEM. ***P < 0.001, according to one-way ANOVA followed by Tukey’s post-hoc test. Scale bar, 10 µm. (**F**,**G**) Autophagic flux indicates differences in the levels of LC3-II of GABARAP-II in the presence and absence of chloroquine (CQ). The bar graphs illustrate the level of LC3-II or GABARAP-II. The levels of LC3-II and GABARAP-II in the GFP- or RavZ(ΔCA)-GFP-expressing cells were normalized to that of actin in HEK293T cells expressing GFP or RavZ(ΔCA)-GFP. The data are presented as the mean ± SEM of five independent experiments. Extended blot images including these data are presented in Supplementary Fig. 5B. RAP, GABARAP.

It has been reported that RavZ protein secreted from Legionella is targeted to autophagic membranes and delipidates mATG8-PE on autophagic membranes in cells^19,20^. Consistent with this, overexpression of 3xFlag-RavZ but not 3xFlag-RavZ_C258S,_ a catalytic mutant of RavZ, reduced LC3B-II in HEK293T Cells (Fig. 1A). These results indicate that RavZ protein is targeted to autophagic membranes and the catalytic domain of RavZ could delipidate mATG8-PE on autophagic membranes. Therefore, deletion of the catalytic domain of RavZ can be used as the target for new autophagic membrane probes. We first deleted the catalytic domain of RavZ and replaced it with GFP to generate RavZ(ΔCA)-GFP (Fig. 1B, upper and Supplemental Fig. 1A). As shown in Fig. 1B (lower), RavZ(ΔCA)-GFP was localized with mRFP-LC3B- or mRFP-GABARAP-positive autophagic membranes in rapamycin/NH_4_Cl-treated mouse embryonic fibroblast (MEF) cells. Quantitative analysis showed that RavZ(ΔCA)-GFP was co-localized with mRFP-LC3B- or mRFP-GABARAP-positive autophagic membranes at similar levels (Fig. 1C). In addition, RavZ(ΔCA)-GFP detected a vesicle structure in wild-type HeLa cells, but not in ATG5- or ATG7-knockout HeLa cells in an autophagy-dependent manner (Supplemental Fig. 2).

Next, we examined whether RavZ(ΔCA)-GFP could detect autolysosomes by using LysoTracker. For this purpose, LysoTracker was added to detect acidic vesicles in MEFs expressing GFP, GFP-LC3B or RavZ(ΔCA)-GFP. As shown in Fig. 1D,E, RavZ(ΔCA)-GFP or GFP-LC3B was localized to LysoTracker-positive autolysosomes in rapamycin-treated MEF cells similarly, suggesting that RavZ(ΔCA)-GFP-positive autophagic membranes were also recruited to autolysosomes as similar with GFP-LC3B positive autophagic membranes.

Because RavZ(ΔCA)-GFP binds to the mATG8 proteins, it may also affect autophagic flux via expression of the exogenous LIR motif. The latter process may stimulate sequestration of endogenous LC3B and/or modify its functions. To measure autophagic flux, the levels of autophagic substrates, such as LC3B-II or GABARAP-II, in HEK293T (rapamycin treatment) cells expressing GFP or RavZ(ΔCA)-GFP in the presence or absence of 50 μM CQ were quantified by western blotting^30,31^. As shown in Fig. 1F,G, the level of substrate proteins, which indicates the autophagic flux rate, in HEK293T cells expressing RavZ(ΔCA)-GFP was similar to that in control cells expressing GFP. This suggests that the expression of RavZ(ΔCA)-GFP sensors did not affect autophagic flux in the turnover assay of endogenous LC3B or GABARAP.

**Figure 2:**
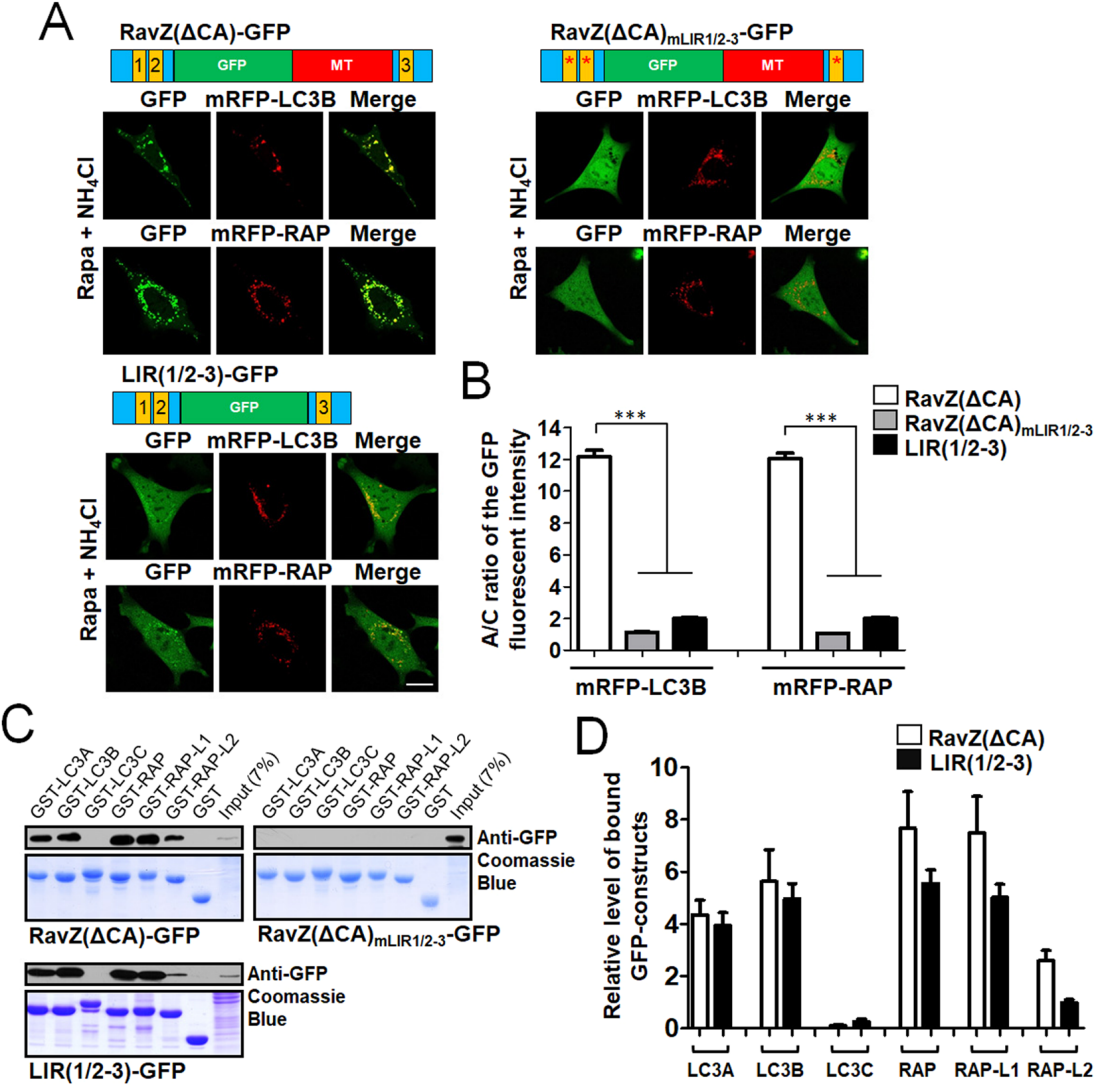
Roles of the LIR motifs of RavZ on mATG8-positive autophagic membrane targeting. (**A**,**B**) Contribution of the LIR motifs of GFP-fused LIR motifs of RavZ in autophagosome targeting. RavZ(ΔCA)_mLIR1/2-3_-GFP, an LIR1/2/3 mutant of RavZ(ΔCA)-GFP; LIR(1/2-3)-GFP, an MT domain deletion mutant of RavZ(ΔCA)-GFP. Confocal images (**A**) depicting the cellular localization of GFP-fused LIR motifs of RavZ in rapamycin (Rapa)/NH_4_Cl-treated MEF cells. Scale bar: 10 μm. The bar graphs (**B**) illustrate the GFP fluorescence intensities of the autophagic membranes and the cytosol (the A/C ratio) (*n* = 75 for each group). ***P < 0.001 (one-way analysis of variance (ANOVA) followed by Tukey’s *post-hoc* test). (**C**,**D**) mATG8 protein-binding properties of the GFP-fused LIR motifs of RavZ proteins using GST-pulldown assays and quantification analysis for the binding. Extended blot images including these data are presented in Supplementary Fig. 6A. The bar graphs (**D**) illustrate relative quantification of the level of bound GFP-constructs in GST-pulldown assay. The levels of bound GFP-constructs intensity were normalized to the intensity of the expressed GFP-constructs (input). The data are presented as the mean ± SEM of three independent experiments. RAP, RAP-L1, GABARAP-L1; RAP-L2, GABARAP-L2.

It has been reported that in RavZ protein, LIR motifs bind directly to mATG8 proteins, whereas an MT domain specifically binds to PI3P on the cytoplasmic surface of the intracellular membrane^19,20^. To further evaluate the contributions of LIR motifs and MT domains within RavZ to the autophagic membrane targeting of RavZ(ΔCA)-GFP, we generated RavZ(ΔCA)_mLIR1/2-3_-GFP, an LIR1/2-3 mutant of RavZ(ΔCA)-GFP, and LIR(1/2-3)-GFP, an MT domain deletion mutant of RavZ(ΔCA)-GFP (Fig. 2A). Each construct was co-expressed with mRFP-LC3 or mRFP-GABARAP proteins in MEF cells. To quantify the autophagic membrane localization, the ratio of GFP fluorescence intensity in autophagic membranes to that of cytosol (the A/C ratio) was measured (Fig. 2B). As shown in Fig. 2A,B, RavZ(ΔCA)-GFP was strongly localized to mRFP-LC3B- or mRFP-GABARAP-positive autophagic membranes, while LIR(1/2-3)-GFP was more weakly localized with mRFP-LC3B- or mRFP-GABARAP-positive autophagic membranes than RavZ(ΔCA)-GFP (***P < 0.001, one-way analysis of variance (ANOVA) followed by Tukey’s *post-hoc* test). However, the LIR mutant RavZ(ΔCA)_mLIR1/2-3_-GFP was not localized to autophagic membranes even in rapamycin/NH_4_Cl-treated MEF cells. Taken together, these results indicate that an MT domain alone, which is known to bind to PI3P, is insufficient for early endosome and autophagic membrane targeting in cells. In addition, the LIR motif alone was weakly targeted to autophagic membranes. However, the combination of an LIR motif and an MT domain resulted in efficient LC3B or GABARAP-positive autophagic membrane targeting.

Next, we performed GST-pulldown assays to elucidate the binding properties of each mutant with mATG8 proteins. As shown in Fig. 2C,D, RavZ(ΔCA)-GFP and LIR(1/2-3)-GFP bound to GST-LC3A/B and GST-GABARAP/-L1/-L2 but not to GST-LC3C at a similar level, whereas RavZ(ΔCA)_mLIR1/2-3_-GFP did not bind to any of the GST-mATG8 proteins tested. Considering the cellular localization and mATG8 protein binding results, LIR motifs are primarily involved in mATG8 binding and an MT domain is additionally required for efficient autophagic membrane targeting via membrane association.

### Characterization of LC3- and GABARAP-binding to the LIR1/2 and LIR3 motifs of RavZ(ΔCA)-GFP

**Figure 3:**
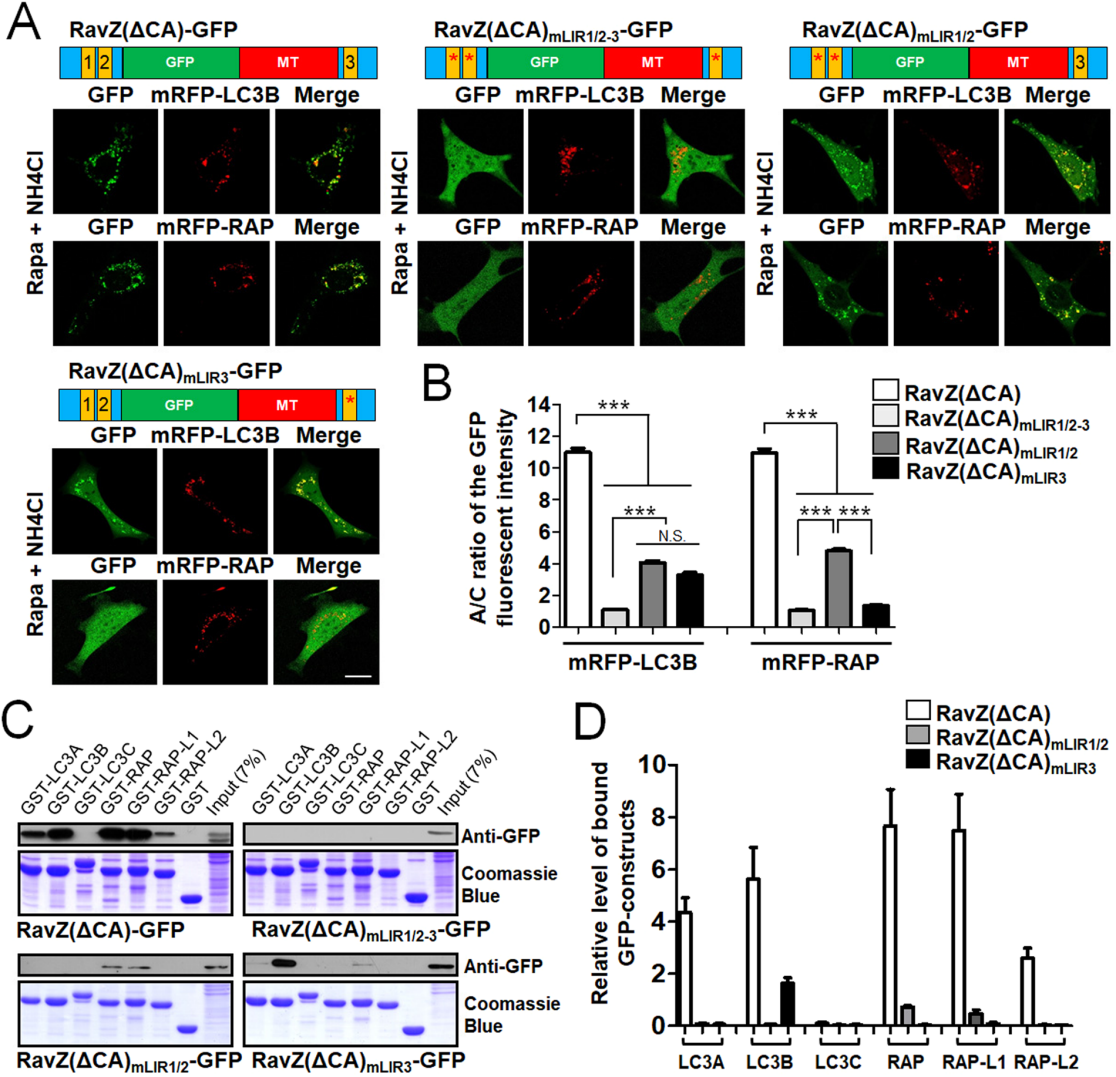
Elucidation of the roles of N- or C-terminal LIR motifs for autophagosome targeting of RavZ(ΔCA)-GFP. (**A**,**B**) Schematic diagram of GFP-fused RavZ mutant proteins and confocal images depicting the cellular localization of GFP-fused LIR motifs in RavZ proteins in MEF cells treated with 100 nM rapamycin (rapa) + 10 mM NH_4_Cl. The bar graphs (**B**) illustrate the GFP fluorescence intensities of the autophagic membranes and the cytosol (the A/C ratio) (*n* = 75 for each group). ***P < 0.001 (*n* = 75 for each group) one-way analysis of variance (ANOVA) followed by Tukey’s *post-hoc* test). Scale bar: 10 μm. (**C**,**D**) mATG8 protein-binding properties of the GFP-fused LIR motifs of RavZ proteins using GST-pulldown assays and quantification analysis for the binding. Extended blot images including these data are presented in Supplementary Fig. 6B. The bar graphs (**D**) illustrate relative quantification of the level of bound GFP-constructs in GST pull-down assay. The levels of bound GFP-constructs intensity were normalized to the intensity of the expressed GFP-constructs (input). The data are presented as the mean ± SEM of three independent experiments. RavZ(ΔCA)_mLIR1/2-3_-GFP, LIR1/2/3 mutant of RavZ(ΔCA)-GFP; RavZ(ΔCA)_mLIR1/2_-GFP, LIR1/2 motif mutant of RavZ(ΔCA)-GFP; RavZ(ΔCA)_mLIR3_-GFP, LIR3 motif mutant of RavZ(ΔCA)-GFP. RAP, GABARAP; RAP-L1, GABARAP-L1; RAP-L2, GABARAP-L2; N.S., not significant.

To further evaluate the differential roles of N-terminal LIR1/2 motifs and the C-terminal LIR3 motif within RavZ, we generated an LIR1/2 motif mutant of RavZ(ΔCA)-GFP (RavZ(ΔCA) _mLIR1/2_-GFP) and an LIR3 motif mutant of RavZ(ΔCA)-GFP (RavZ(ΔCA)_mLIR3_-GFP). As shown in Fig. 3A,B, RavZ(ΔCA)_mLIR1/2_-GFP was localized with mRFP-LC3B- or mRFP-GABARAP-positive autophagic membranes, albeit less efficiently than RavZ(ΔCA)-GFP (***P < 0.001, one-way analysis of variance (ANOVA) followed by Tukey’s *post-hoc* test). On the other hand, RavZ(ΔCA)_mLIR3_-GFP was localized with mRFP-LC3B-positive autophagic membranes, but not with mRFP-GABARAP-positive autophagic membranes. These results suggest that both LIR1/2 motifs and LIR3 motif are involved in autophagic membrane targeting of RavZ(ΔCA)-GFP.

Next, we performed GST-pulldown assays to elucidate the binding properties of each mutant with the mATG8 protein family. As shown in Fig. 3C,D, RavZ(ΔCA)_mLIR1/2_-GFP bound to GST-GABARAP/-L1 but not to GST-LC3A/B/C and GST-GABARAP-L2, while RavZ(ΔCA)_mLIR3_-GFP bound to GST-LC3B and weakly to GST-GABARAP-L1. As a control, RavZ(ΔCA)-GFP, but not RavZ(ΔCA)_mLIR1/2-3_-GFP bound to GST-LC3A/B and GST-GABARAP/-L1/-L2 but not to GST-LC3C. Quantification of GST binding showed that the binding of any mATG8 to RavZ(ΔCA)-GFP was higher than the binding to either RavZ(ΔCA)_mLIR1/2_-GFP or RavZ(ΔCA)_mLIR3_-GFP (Fig. 3D), indicating that both the LIR1/2 and LIR3 motifs contributed to the LC3 or GABARAP subfamily binding of RavZ protein. Overall, the results of both cellular localization and GST-pulldown assays suggest that both the LIR1/2 and LIR3 motifs are required for mATG8 proteins binding except for LC3C binding.

Our cellular analysis of the modified RavZ(ΔCA)-GFP indicated that the protein is efficiently targeted to autophagosomes via a combination of LIR motifs and an MT domain. An MT domain, an LIR1/2 motif, or an LIR3 motif alone is negligible or weak for targeting RavZ to autophagic membranes. However, when the three domains are combined, as in the RavZ(ΔCA)-GFP protein, the protein is efficiently targeted to autophagic membranes. It has been reported that the catalytic domain of the RavZ protein also has an *α3* helix, which is involved in the association of the membrane with enzyme activity (Supplemental Fig. 1A)^20^. Therefore, in wild-type RavZ, efficient autophagic membrane targeting of RavZ protein is mediated by the combination of multiple domains including two membrane association domains (an *α3* helix in the catalytic domain and a PI3P-binding MT domain) and multiple LIR motifs (an LIR1/2 motifs and an LIR3 motif) for direct mATG8 protein binding.

### Comparative analysis of the autophagosome targeting efficiency between HyD motifs and MT domains

**Figure 4:**
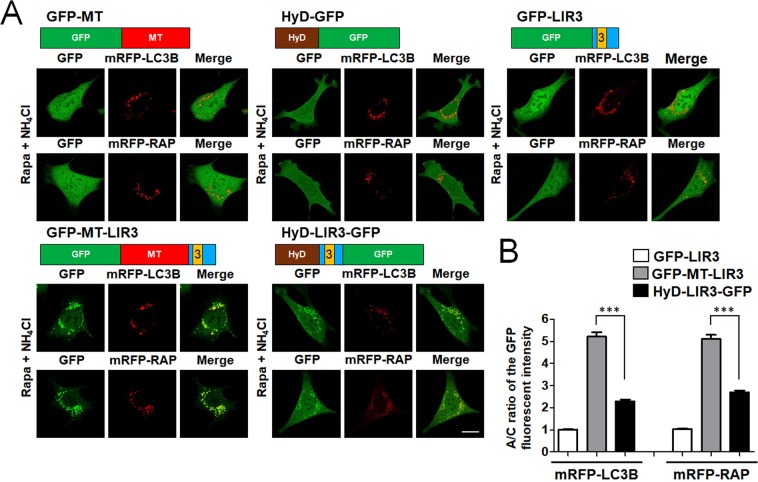
Comparison of the autophagosome targeting efficiency of HyD and MT domains. (**A**) Schematic diagram of GFP-fused RavZ mutant proteins and confocal images showing cellular localization of mRFP-LC3B or mRFP-GABARAP co-expressed with GFP-LIR3 or its derivative forms in MEF cells upon autophagy induction (100 nM rapamycin (rapa) + 10 mM NH_4_Cl, 3 h). Scale bar: 10 μm. The bar graphs (**B**) illustrate the GFP fluorescent intensities of the autophagosomes and the cytosol (the A/C ratio) (*n* = 75 for each group). GFP-MT, GFP fused to an MT domain; GFP-LIR3, an LIR3 motif from RavZ fused to GFP; GFP-MT-LIR3, an LIR3 motif from RavZ fused to GFP-MT; HyD-LIR3-GFP, an LIR3 motif from RavZ fused to HyD-GFP. ***P < 0.001 (one-way analysis of variance (ANOVA) followed by Tukey’s *post-hoc* test). mRFP-RAP, mRFP-GABARAP.

We recently developed LIR-based autophagosome sensors using a HyD motif, which enhances the membrane localization^15^. In RavZ proteins, an MT domain, another type of membrane association domain, plays an assisting role in autophagosome targeting of RavZ(ΔCA)-GFP (Fig. 2). Therefore, to compare the relative efficiency of autophagosome targeting between a HyD motif and an MT domain, we generated several constructs, as shown in Fig. 4A: GFP fused to an MT domain (GFP-MT) and to an LIR3 motif from RavZ fused to GFP, GFP-MT, or HyD-GFP (GFP-LIR3, GFP-MT-LIR3, and HyD-LIR3-GFP, respectively). Each construct was co-expressed with either mRFP-LC3B or mRFP-GABARAP in MEF cells. As shown in Fig. 4A, GFP-LIR3, GFP-HyD, or GFP-MT alone was diffusely localized to the cytosol and nucleus and was barely localized to autophagic membranes. However, GFP-MT-LIR3 and HyD-LIR3-GFP were co-localized with mRFP-LC3B- or mRFP-GABARAP-positive autophagic membranes in rapamycin/NH_4_Cl-treated cells (Fig. 4A,B). Thus, when LIR motifs were combined with an MT domain or a HyD motif, the A/C ratio was significantly enhanced. More intriguingly, GFP-MT-LIR3 was more efficiently localized to autophagic membranes than HyD-LIR3-GFP (***P < 0.001, one-way analysis of variance (ANOVA) followed by Tukey’s *post-hoc* test). Therefore, our comparative analysis of the A/C ratios between MT- and HyD-LIR probes suggests that an MT domain facilitates autophagic membrane-targeting through PI3P-binding more efficiently than a HyD motif through non-selective membrane association via hydrophobic interactions on autophagic membranes.

### Monitoring LC3 or GABARAP subfamily-positive autophagic membranes using RavZ-based probes modified by replacement of LIR motifs with those selective for members of the LC3 or GABARAP subfamily

**Figure 5:**
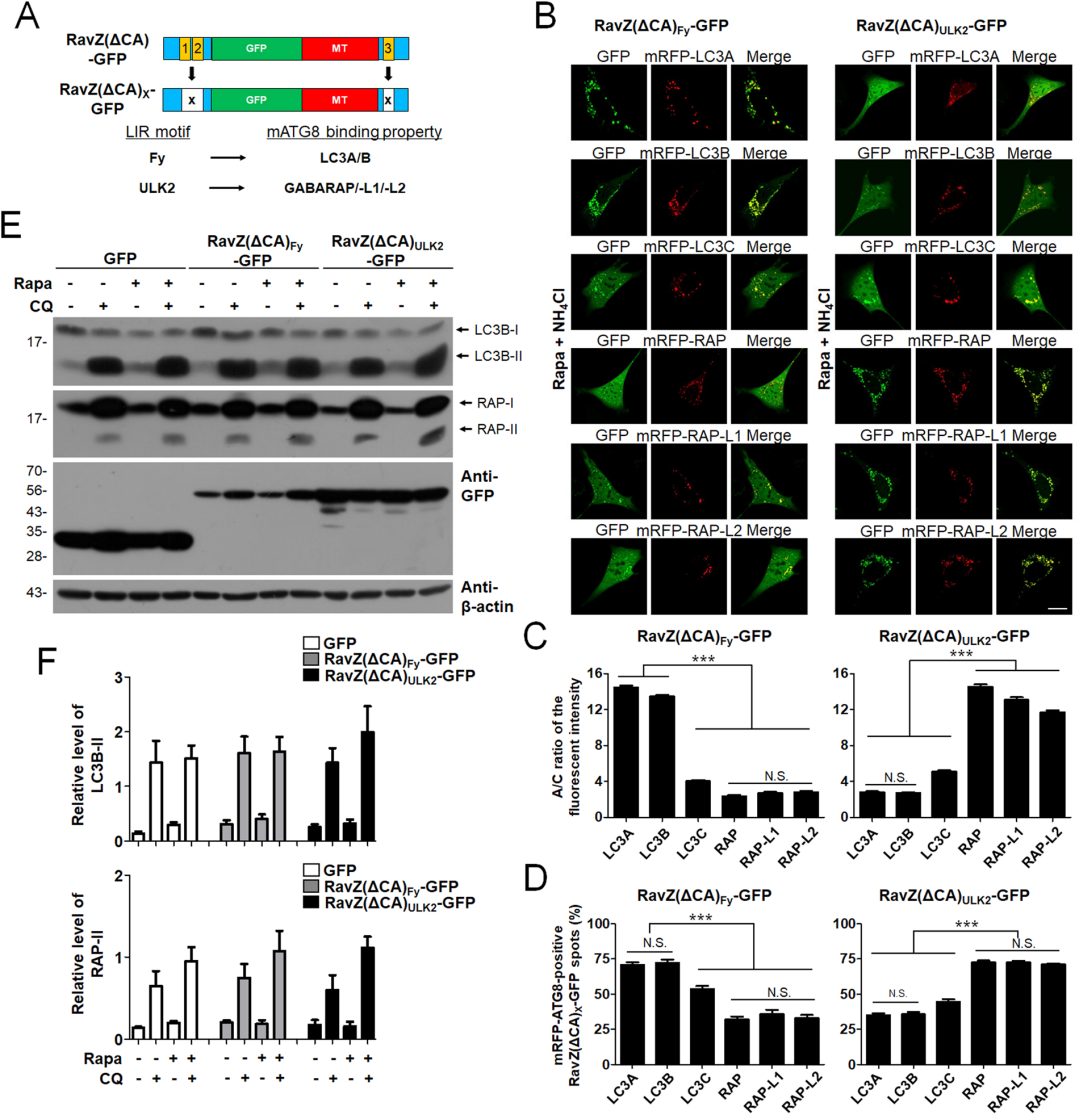
Selective LC3A/B or GABARAP subfamily-positive autophagosome targeting of RavZ(ΔCA)_Fy_-GFP or RavZ(ΔCA)_ULK2_-GFP, respectively. (**A**) Schematic diagram of GFP-fused RavZ(ΔCA)_X_-GFP and its binding preference. (**B**) Confocal images showing the cellular localization of RavZ(ΔCA)_Fy_-GFP or RavZ(ΔCA)_ULK2_-GFP in rapamycin/NH_4_Cl-treated MEF cells. Scale bar: 10 μm. The bar graphs (**C**) illustrate the GFP fluorescent intensities of the autophagosomes and the cytosol (the A/C ratio) (*n* = 75 for each group). (**D**) Percentage of co-localization of mRFP-mATG8-positive autophagic membrane with RavZ(ΔCA)_Fy_-GFP or RavZ(ΔCA)_ULK2_-GFP-positive autophagic membranes (*n* = 25 for each group). (**E**,**F**) Autophagic flux assay in HEK293T cells expressing GFP, RavZ(ΔCA)_Fy_-GFP, or RavZ(ΔCA)_ULK2_-GFP upon rapamycin (rapa) treatment (in the presence or absence of chloroquine (CQ) for 3 h). The bar graphs illustrate the level of LC3-II or GABARAP-II. The levels of LC3-II and GABARAP-II in the GFP, RavZ(ΔCA)_Fy_-GFP, or RavZ(ΔCA)_ULK2_-GFP-expressing cells were normalized to that of actin in HEK293T cells expressing GFP, RavZ(ΔCA)_Fy_-GFP, or RavZ(ΔCA)_ULK2_-GFP-expressing cells. The cell lysates were then subjected to western blot analyses (**E**) and quantification analysis (**F**) with an anti-GFP, anti-LC3, anti-GABARAP, or anti-β-actin antibody. The data are presented as the mean ± SEM of five independent experiments. Extended blot images including these data are presented in Supplementary Fig. 7. RAP, GABARAP; RAP-L1, GABARAP-L1; RAP-L2, GABARAP-L2; N.S., not significant.

If RavZ(ΔCA)-GFP constructs are to be used for monitoring LC3- or GABARAP-positive autophagic membranes selectively, the LIR motifs of RavZ must be replaced with other LIR motifs that specifically and selectively bind to LC3 or GABARAP proteins. Based on previous studies that analyzed the preferential binding properties of LIR motifs for either LC3 or GABARAP, we chose candidate LIR motifs from Fyco1 as an LC3 subfamily-specific motif and from ULK2 as GABARAP subfamily-specific motifs (Fig. 5A). To generate selective LC3- or GABARAP-positive autophagic membrane-detecting RavZ-based probes, we replaced the LIR1/2 and LIR3 motifs within RavZ(ΔCA)-GFP with LIR motifs from Fyco1 or ULK2, generating RavZ(ΔCA)_Fy_-GFP and RavZ(ΔCA)_ULK2_-GFP, respectively. Each probe, which contains two LIR motifs and an MT domain, was co-expressed with each 3xFlag-mATG8 protein in HEK293T cells and Flag co-immunoprecipitation (Flag co-IP) assays were performed to investigate the binding properties of these new LIR motifs. As shown in Supplemental Fig. 3, RavZ(ΔCA)_Fy_-GFP bound selectively to 3xFlag-LC3A or 3xFlag-LC3B, but not to 3xFlag-LC3C or 3xFlag-GABARAP, -L1, or -L2. In contrast, RavZ(ΔCA)_ULK2_-GFP bound to 3xFlag-GABARAP, -L1, or -L2 but not to 3xFlag-LC3A, B, or C.

Next, RavZ(ΔCA)_Fy_-GFP or RavZ(ΔCA)_ULK2_-GFP was co-expressed with each of the mRFP-mATG8 proteins in MEF cells. As shown in Fig. 5B,C, RavZ(ΔCA)_Fy_-GFP was efficiently localized to mRFP-LC3A/B-positive autophagosomes, whereas RavZ(ΔCA)_ULK2_-GFP was efficiently localized to mRFP-GABARAP/-L1/-L2-positive autophagosomes in MEF cells (***P < 0.001, one-way analysis of variance (ANOVA) followed by Tukey’s *post-hoc* test). Similarly, RavZ(ΔCA)_Fy_-GFP-positive spots were more co-localized with mRFP-LC3 subfamily-positive spots than mRFP-GABARAP subfamily-positive spots (***P < 0.001, one-way analysis of variance (ANOVA) followed by Tukey’s *post-hoc* test) (Fig. 5D). On the other hand, RavZ(ΔCA)_ULK2_-GFP-positive spots were more co-localized with mRFP-GABARAP subfamily-positive spots than mRFP-LC3 subfamily-positive spots (***P < 0.001, one-way analysis of variance (ANOVA) followed by Tukey’s *post-hoc* test) (Fig. 5D). In addition, RavZ(ΔCA)_Fy_-GFP and RavZ(ΔCA)_ULK2_-GFP detected vesicle structures in wild-type HeLa cells, but not in ATG5- or ATG7-knockout HeLa cells in an autophagy-dependent manner (Supplemental Fig. 2).

As shown in Fig. 5E,F, the expression of RavZ(ΔCA)_Fy_-GFP or RavZ(ΔCA)_ULK2_-GFP did not affect autophagic flux in the turnover assay of endogenous LC3B or GABARAP. These results suggest that RavZ(ΔCA)_Fy_-GFP and RavZ(ΔCA)_ULK2_-GFP can detect endogenous autophagic membranes and preferentially detect LC3A/B-positive and GABARAP-positive autophagic membranes, respectively.

Next, we examined the dynamics of RavZ(ΔCA)_X_-GFP-positive autophagic membranes in MEF cells using live-cell imaging. Kymograph analysis of GFP-LC3B, GFP-GABARAP, RavZ(ΔCA)-GFP RavZ(ΔCA)_Fy_-GFP, and RavZ(ΔCA)_ULK2_-GFP mobility showed no difference between the groups (Supplemental Fig. 4).

We summarized the A/C ratio of the constructs used in the experiments in Supplemental Table 1. The A/C ratio clearly showed that RavZ(ΔCA)-GFP efficiently detected both LCB and GABARAP-positive autophagic membranes. Meanwhile, RavZ(ΔCA)_Fy_-GFP selectively detected LC3A/B-positive autophagic membranes, whereas RavZ(ΔCA)_ULK2_-GFP efficiently detected GABARAP subfamily-positive autophagic membranes. RavZ(ΔCA)_mLIR3_-GFP also selectively detected LC3B-positive autophagic membranes but to a much weaker degree than RavZ(ΔCA)_Fy_-GFP. RavZ(ΔCA)_mLIR1/2_-GFP detected both LC3B- and GABARAP-positive autophagic membranes to a much weaker degree than RavZ(ΔCA)-GFP. Thus, RavZ(ΔCA)-GFP, RavZ(ΔCA)_Fy_-GFP, and RavZ(ΔCA)_ULK2_-GFP are useful for detecting all types of mATG8-positive, LC3A/B-positive, and GABARAP subfamily-positive autophagic membranes, respectively.

Many mATG8-interacting proteins contain a canonical LIR motif with a core consensus sequence, (W/F/Y)-X-X-(L/I/V), which binds to LIR docking sites (LDS) in two hydrophobic pockets, HP1 and HP2, conserved in mATG8s using W/F/Y and L/I/V, respectively^9,32–35^. As shown in Supplemental Fig. 1B, canonical LIR motifs from RavZ, Fyco1, and ULK2 have “F” in a core LIR motifs commonly. Recently, the GABARAP-selective motif (GIM) was proposed to have a core consensus sequence ((W/F)-(I/V)-X-V)^36^. The LIR motif from ULK2 has “FVLV,” which follows the GIM consensus sequence. The LIR3 motif of RavZ has “FVTI,” which is similar to the GIM sequence ((W/F)-(I/V)-X-V) except for the presence of “I” instead of “V”. This might be the reason why the LIR3 motif of RavZ has GABARAP-preferential binding.

In a previous study, we used a general membrane association motif, a HyD motif, to monitor endogenous mATG8 family proteins in autophagosomes in live cells without overexpression^15,21^. HyD motifs have mild hydrophobicity and, by themselves, have no organelle membrane targeting; instead, these motifs help enhance membrane association mediated by LIR motifs^15^. Similarly, we found that the MT domain of RavZ alone is localized to the cytosol, but not to the early endosome, where it fails to enrich PI3P probably due to weak PI3P binding, but helps to enhance membrane association mediated by LIR motifs (Figs 2A,B and 4). Therefore, using an MT domain of RavZ can minimize the sequestering and altering of PI3P dynamics in cells. In a previous study, we duplicated LIR motifs to enhance the efficiency of autophagic membranes further and generated GABARAP subfamily-positive autophagic membrane-targeting probes (HyD-2xLIR(ULK2)-GFP and HyD-2xLIR(Stbd1)-GFP)^21^. Interestingly, the wild-type RavZ protein has multiple LIR motifs that enhance autophagic membrane targeting through the synergistic binding avidity of the N- and C-terminal LIR motifs. Thus, RavZ has a useful structure for sensing autophagic membranes. We leveraged this advantage in our study to generate probes that detect LC3A/B- or GABARAP-positive autophagic membranes in cells. However, if LIR-based sensors are expressed at a higher level, they could potentially function as dominant-negative probes that sequester endogenous LIR-containing proteins or PI3P. Therefore, to be used as probes to detect LC3- or GABARAP-positive autophagic membranes, stable cell lines that express RavZ(ΔCA)_X_-GFP at a lower level or promoters that mediate lower expression levels need to be considered. Despite some limitations, we propose that RavZ(ΔCA)_X_-GFP constructs are an advanced version of LIR-based LC3- or GABARAP-positive autophagic membrane-detecting probes for autophagy research.

## Methods

### DNA constructs

All primers are listed in Supplemental Table 2. The regions encoding individual RavZ LIR1/2 or LIR3 motifs and MT domains were generated by PCR amplification of pcDNA3.1(−)-Flag-RavZ vectors and inserted into the N3-EGFP vector using restriction enzymes. The pcDNA3.1(−)-Flag-RavZ vectors were kindly provided by Dr. Song (Department of Life Sciences, Korea University, Korea)^19^. The *Aplysia* PDE4 short-form (N20) (SN20, HyD)-GFP was generated by PCR amplification of the full-length Aplysia PDE4 short-form gene and inserted into the pcDNA3.1-EGFP and N3-EGFP vectors. Mutations of the RavZ LIR motif were amplified by PCR using RavZ LIR1/2 or 3 mutant primers (Supplemental Table 2) and inserted into N3-RavZ-EGFP vectors using restriction enzymes. Additionally, other LIR motifs, including FUNDC1, Fyco1, Stbd1, and ULK2, were amplified by PCR using primers (Supplemental Table 2) and inserted into N3-RavZ-GFP vectors to replace the RavZ LIR with another LIR. GST-LC3A, GST-LC3B, GST-LC3C, GST-GABARAP, GST-GABARAP-L1, and GST-GABARAP-L2 were obtained from Addgene (Cambridge, MA, USA). We also used previously described DNA constructs mRFP-LC3A, mRFP-LC3B, mRFP-LC3C, mRFP-GABARAP, mRFP-GABARAP-L1, and mRFP-GABARAP-L2^15^ in this study.

### Cell culture, transfection, confocal microscopy, and drug treatment

This method has been previously described^37^. Briefly, HEK293T, MEF, and HeLa cells were cultured in Dulbecco’s modified Eagle’s medium (DMEM) supplemented with 10% (v/v) fetal bovine serum and penicillin/streptomycin in a humidified atmosphere of 5% (v/v) CO_2_ at 37 °C. Cells were seeded in a Sticky-Slide 8-well system (Catalog #: 80828; Ibidi, Martinsried, Germany) to obtain 40–60% confluent cells on the day of imaging. Cells were transfected with DNA constructs using calcium phosphate or Lipofectamine 2000 (Life Technologies, Carlsbad, CA, USA) 20–24 h before imaging. The relative amount of each construct was empirically determined based on the relative expression of each construct combination.

Cells were visualized with an inverted Zeiss LSM-700 scanning laser confocal microscope operated by ZEN software (Carl Zeiss, Oberkochen, Germany). The laser lines for excitation and the spectral detection windows for the fluorochromes were 488 with 508–543 nm for GFP and 561 with 578–649 nm for mRFP. Appropriate GFP (500–550 nm) and mRFP (575–625 nm) emission filters were used during the sequential imaging of each fluorescent protein. Most images were taken with live cells. Rapamycin was obtained from Sigma-Aldrich (Catalog #: R8781; St. Louis, MO, USA). All treatments and assays were performed at 37 °C unless otherwise indicated.

### Quantitative analysis of A/C ratio

To calculate the ratio of autophagosome/cytosol (A/C) fluorescent intensities, the average value of the autophagosome or cytosol fluorescent intensity was obtained from at least five randomly selected points on autophagosomes or in the cytosol of a single cell using ZEN software. In the same manner, the quantitative A/C ratio of at least 25 randomly selected cells per experiment was obtained from three independent experiments. All statistical data were calculated and graphed using GraphPad Prism5 (GraphPad, Inc., La Jolla, CA, USA).

### Co-localized spot number analysis

To determine the percent of co-localized spots of LC3/GABARAP-positive autophagosomes in autophagy-induced cells, the number of co-localized spots over a certain size in a single cell was counted using Image-J software. First, the cell image was changed to an 8-bit image and then inverted. Next, the background was removed so that only the spot was visible, and finally, the number of co-localized spots was counted using the “Analyze particles” function in the Image-J program. In the same manner, at least 25 randomly selected cells were quantified. All statistical data were calculated and graphed using GraphPad Prism5.

### GST-pulldown assay

For GST-pulldown assays using HEK293T cell lysates, cells were transfected with the GFP construct-containing DNA using calcium phosphate (Takara Bio) transfection. After 24 h, cells were harvested, washed with PBS, and lysed in immunoprecipitation lysis buffer solution (50 mM Tris-HCl, pH 7.5; 150 mM NaCl; 2 mM EDTA; 1% Triton X ‐100; and protease and phosphatase inhibitors), and the supernatants were isolated after centrifugation. Cell lysates were incubated with purified GST-mATG8 protein and glutathione-agarose beads overnight at 4 °C. The next day, they were washed 3–5 times with immunoprecipitation lysis buffer solution at 4 °C. Proteins were separated by SDS–PAGE and analyzed by Western blot and Coomassie blue staining.

### Immunoprecipitation

This method has been previously described^38^. Briefly, for transient transfections, HEK293T cells were plated at a density of 5–7 × 10^5^ cells/well in six-well plates and cultured for 24 h. The cells were transfected with DNA constructs using calcium phosphate (Clontech) and incubated for 24 h. For Flag immunoprecipitation, the transfected HEK293T cells were washed twice with PBS and lysed with a buffer containing 1% Triton X-100, 50 mM Tris-HCl (pH 7.5), 150 mM sodium chloride (NaCl), 2 mM ethylenediaminetetraacetic acid (EDTA), and a protease inhibitor cocktail (Roche). The cell lysate was incubated with 50 μL (bead volume) of mouse anti-Flag M2 antibody-conjugated beads (Sigma) at 4 °C overnight. The beads were subsequently washed three times with lysis buffer. The immunoprecipitate was eluted by adding 2 μg/mL of 3xFlag peptides and analyzed by Western blot.

### Western blot, antibodies and band quantitation

Protein samples from the GST-pulldown, immunoprecipitation assays, and flux assays were separated by SDS-PAGE, transferred to PVDF membranes, and incubated with primary antibodies overnight at 4 °C. After three washes, membranes were incubated with secondary antibodies and conjugated with horseradish peroxidase for an hour. Signals were visualized with ECL using Advansta WesternBright ECL (K-12045-D50). The antibodies using in the experiment were used: Flag (Sigma, F1804, 1:10000), GFP (Santa Cruz Biotechnology, sc-9996, 1:10000), LC3B (Cell Signaling Technology, #2775, 1:1000), GABARAP (Cell Signaling Technology, #13733, 1:1000), donkey anti-rabbit HRP (Santa Cruz Biotechnology, sc-2313, 1:10000) and goat anti-mouse HRP (Santa Cruz Biotechnology, sc-2005, 1:10000). In order to quantify the intensity of the western blot band, the area of each band was quantified using the ImageJ program. In the same manner, the Band Quantitation was obtained from three independent experiments. All statistical data were calculated and graphed using GraphPad Prism5 (GraphPad, Inc., La Jolla, CA, USA).

### Live cell imaging and autophagosome dynamics analysis

MEFs were transfected using Lipofectamine 2000 and expressed for 24 h on 96-well glass-bottom plates (Ibidi, #89626). Before analysis, the cells were incubated with rapamycin (100 nM, 4 h) to induce autophagy. Images of autophagosome dynamics were acquired on an A1R confocal microscope (Nikon, Japan) with a Nikon CFI Plan Apochromat VC object (60x/1.40 numerical aperture) in a temperature-controlled chamber at 37 °C. A 525-nm laser was used for excitation. The images were captured every 5 s for 5 min for GFP-LC3, GFP-GABARAP, RavZ(ΔCat)-GFP, RavZ(ΔCat, Fyco1)-GFP, and RavZ(ΔCat, ULK2)-GFP. Kymograph images and movies were generated using ImageJ (NIH) software to compare the dynamics of autophagosomes. Autophagosome dynamics was analyzed using NIS-elements AR analysis program (Nikon).

### Statistical analysis

Kolmogorov-Smirnov (KS) tests were used to examine the distribution of the data. The data were normally distributed, and then one-way ANOVA, in conjunction with Tukey’s multiple comparison test for *post-hoc* analysis (group number > = 3) was used for statistical analysis.

## Supplementary information

Supplementary information

## References

[1] Kimmey, J. M. & Stallings, C. L. Bacterial Pathogens versus Autophagy: Implications for Therapeutic Interventions. *Trends Mol Med* **22**, 1060–1076, doi:S1471-4914(16)30150-2 (2016). 10.1016/j.molmed.2016.10.008 PMC5215815 27866924

[2] Sharma V, Verma S, Seranova E, Sarkar S, Kumar D (2018). Selective Autophagy and Xenophagy in Infection and Disease. Front Cell Dev Biol.

[3] Sorbara, M. T. & Girardin, S. E. Emerging themes in bacterial autophagy. *Curr Opin Microbiol* **23**, 163–170, doi:S1369-5274(14)00190-8 (2015). 10.1016/j.mib.2014.11.020 25497773

[4] Kwon DH, Song HK (2018). A Structural View of Xenophagy, a Battle between Host and Microbes. Molecules and cells.

[5] Ohsumi Y (2001). Molecular dissection of autophagy: two ubiquitin-like systems. Nat Rev Mol Cell Biol.

[6] Nakatogawa, H., Ichimura, Y. & Ohsumi, Y. Atg8, a ubiquitin-like protein required for autophagosome formation, mediates membrane tethering and hemifusion. *Cell* **130**, 165–178, doi:S0092-8674(07)00658-7 (2007). 10.1016/j.cell.2007.05.021 17632063

[7] Kabeya Y (2000). LC3, a mammalian homologue of yeast Apg8p, is localized in autophagosome membranes after processing. The EMBO journal.

[8] Kabeya Y (2004). LC3, GABARAP and GATE16 localize to autophagosomal membrane depending on form-II formation. Journal of cell science.

[9] Kalvari, I. *et al*. iLIR: A web resource for prediction of Atg8-family interacting proteins. *Autophagy* **10**, 913–925, doi:28260 (2014). 10.4161/auto.28260 PMC5119064 24589857

[10] Slobodkin, M. R. & Elazar, Z. The Atg8 family: multifunctional ubiquitin-like key regulators of autophagy. *Essays Biochem* **55**, 51–64, doi:bse0550051 (2013). 10.1042/bse0550051 24070471

[11] Tanida I, Ueno T, Kominami E (2004). LC3 conjugation system in mammalian autophagy. Int J Biochem Cell Biol.

[12] Lee, Y. K. & Lee, J. A. Role of the Mammalian ATG8/LC3 Family in Autophagy: Differential and Compensatory Roles in the Spatiotemporal Regulation of Autophagy. *BMB reports*, doi:3562 [pii] (2016). 10.5483/BMBRep.2016.49.8.081 PMC5070729 27418283

[13] Schaaf, M. B., Keulers, T. G., Vooijs, M. A. & Rouschop, K. M. LC3/GABARAP family proteins: autophagy-(un)related functions. *FASEB journal: official publication of the Federation of American Societies for Experimental Biology* **30**, 3961–3978, doi:fj.201600698R (2016). 10.1096/fj.201600698R 27601442

[14] Weidberg, H. *et al*. LC3 and GATE-16/GABARAP subfamilies are both essential yet act differently in autophagosome biogenesis. *The EMBO journal* **29**, 1792–1802, doi:emboj201074 (2010). 10.1038/emboj.2010.74 PMC2885923 20418806

[15] Lee YK (2017). Development of LC3/GABARAP sensors containing a LIR and a hydrophobic domain to monitor autophagy. The EMBO journal.

[16] Stolz, A. *et al*. Fluorescence-based ATG8 sensors monitor localization and function of LC3/GABARAP proteins. *The EMBO journal* **36**, 549–564, doi:embj.201695063 (2017). 10.15252/embj.201695063 PMC5437816 28028054

[17] Choy, A. *et al*. The Legionella effector RavZ inhibits host autophagy through irreversible Atg8 deconjugation. *Science* **338**, 1072–1076, doi:science.1227026 (2012). 10.1126/science.1227026 PMC3682818 23112293

[18] Pantoom S, Yang A, Wu YW (2017). Lift and cut: Anti-host autophagy mechanism of Legionella pneumophila. Autophagy.

[19] Kwon DH (2017). The 1:2 complex between RavZ and LC3 reveals a mechanism for deconjugation of LC3 on the phagophore membrane. Autophagy.

[20] Horenkamp, F. A. *et al*. The Legionella Anti-autophagy Effector RavZ Targets the Autophagosome via PI3P- and Curvature-Sensing Motifs. *Dev Cell* **34**, 569–576, doi:S1534-5807(15)00524-9 (2015). 10.1016/j.devcel.2015.08.010 PMC4594837 26343456

[21] Jeon P (2019). Development of GABARAP family protein-sensitive LIR-based probes for neuronal autophagy. Molecular brain.

[22] Zientara-Rytter K, Subramani S (2018). AIM/LIR-based fluorescent sensors-new tools to monitor mAtg8 functions. Autophagy.

[23] Pankiv, S. *et al*. FYCO1 is a Rab7 effector that binds to LC3 and PI3P to mediate microtubule plus end-directed vesicle transport. *J Cell Biol* **188**, 253–269, doi:jcb.200907015 (2010). 10.1083/jcb.200907015 PMC2812517 20100911

[24] Walker, S., Chandra, P., Manifava, M., Axe, E. & Ktistakis, N. T. Making autophagosomes: localized synthesis of phosphatidylinositol 3-phosphate holds the clue. *Autophagy* **4**, 1093–1096, doi:7141 (2008). 10.4161/auto.7141 18927492

[25] Roberts, R. & Ktistakis, N. T. Omegasomes: PI3P platforms that manufacture autophagosomes. *Essays Biochem* **55**, 17–27, doi:bse0550017 (2013). 10.1042/bse0550017 24070468

[26] Nascimbeni, A. C. *et al*. ER-plasma membrane contact sites contribute to autophagosome biogenesis by regulation of local PI3P synthesis. *EMBO J* **36**, 2018–2033, doi:embj.201797006 (2017). 10.15252/embj.201797006 PMC5509996 28550152

[27] Nascimbeni AC, Codogno P, Morel E (2017). Phosphatidylinositol-3-phosphate in the regulation of autophagy membrane dynamics. The FEBS journal.

[28] Jang DJ, Lee JA (2016). The roles of phosphoinositides in mammalian autophagy. Archives of pharmacal research.

[29] Marat AL, Haucke V (2016). Phosphatidylinositol 3-phosphates-at the interface between cell signalling and membrane traffic. The EMBO journal.

[30] Klionsky DJ (2012). Guidelines for the use and interpretation of assays for monitoring autophagy. Autophagy.

[31] Chittaranjan, S., Bortnik, S. & Gorski, S. M. Monitoring Autophagic Flux by Using Lysosomal Inhibitors and Western Blotting of Endogenous MAP1LC3B. *Cold Spring Harb Protoc* **2015**, pdb prot086256, doi:2015/8/pdb.prot086256 (2015). 10.1101/pdb.prot086256 26240408

[32] Wild, P., McEwan, D. G. & Dikic, I. The LC3 interactome at a glance. *Journal of cell science* **127**, 3–9, doi:jcs.140426 (2014). 10.1242/jcs.140426 24345374

[33] Birgisdottir AB, Lamark T, Johansen T (2013). The LIR motif - crucial for selective autophagy. Journal of cell science.

[34] Ichimura, Y. *et al*. Structural basis for sorting mechanism of p62 in selective autophagy. *The Journal of biological chemistry* **283**, 22847–22857, doi:M802182200 (2008). 10.1074/jbc.M802182200 18524774

[35] Noda, N. N., Ohsumi, Y. & Inagaki, F. Atg8-family interacting motif crucial for selective autophagy. *Febs Lett* **584**, 1379–1385, doi:S0014-5793(10)00037-2 (2010). 10.1016/j.febslet.2010.01.018 20083108

[36] Rogov, V. V. *et al*. Structural and functional analysis of the GABARAP interaction motif (GIM). **18**, 1382–1396, 10.15252/embr.201643587 (2017). 10.15252/embr.201643587 PMC5538626 28655748

[37] Jang, D. J. *et al*. Activation of Aplysia ARF6 induces neurite outgrowth and is sequestered by the overexpression of the PH domain of Aplysia Sec. 7 proteins. *Neurobiol Learn Mem*, doi:S1074-7427(16)30092-2 (2016). 10.1016/j.nlm.2016.06.017 27344941

[38] Kim, K. H. *et al*. Intracellular membrane association of the Aplysia cAMP phosphodiesterase long and short forms via different targeting mechanisms. *The Journal of biological chemistry* **289**, 25797–25811, doi:M114.572222 (2014). 10.1074/jbc.M114.572222 PMC4162181 25077971

