# Supplementary material for: Monitoring LC3- or GABARAP-positive autophagic membranes using modified RavZ-based probes
Source: Sci Rep. 2019 Nov 12;9:16593. doi: 10.1038/s41598-019-53372-2 (PMC6851389; doi:10.1038/s41598-019-53372-2)
Supplement: Supplementary file 1 — Supplementary information [file 41598_2019_53372_MOESM1_ESM.pdf]

Monitoring LC3- or GABARAP-positive autophagic membranes using modified RavZ-based probes

Sang-Won Park[1,4], Pureum Jeon[2,4], Yong-Woo Jun[1], Ju-Hui Park[1], Seung-Hwan Lee, Sangkyu Lee[3], Jin-A Lee[2,*] and Deok-Jin Jang[1,*]

[1]Department of Ecological Science, College of Ecology and Environment, Kyungpook National University, 2559, Gyeongsang-daero, Sangju-si, Gyeongsangbuk-do, 37224, Republic of Korea. [2]Department of Biological Science and Biotechnology, College of Life Science and Nano Technology, Hannam University, 1646, Yuseong-daero, Yuseong-gu, Daejeon 34054, Republic of Korea. [3]Center for Cognition and Sociality, Institute for Basic Science (IBS), Daejeon 34126, Republic of Korea.

[4]These authors contributed equally

* Corresponding author

- Deok-Jin Jang, Ph.D.

2559, Gyeongsang-daero, Sangju-Si, Gyeongsangbuk-do, 37224, Republic of Korea.

E-mail address: jangdj@knu.ac.kr,

Tel: +82-54-530-1213, Fax: +82-54-530-1218,

- Jin-A Lee, Ph.D.

1646, Yuseong-daero, Yuseong-gu, Daejeon 34054, Republic of Korea
E-mail address: leeja@hnu.kr

Phone: +82-42-629-8785; Fax: +82-42-629-8789

A

RavZ full sequence

1 MKGKLTGKDKLIVDEFEELGEQESDIDEFDLLEGDEKLPGDSELDKTTSIYPPETSWEVNKGMNSSRLHKLYSLFFDK
LIR1 LIR2

79 SSAFYLGDDVSVLEDKPLTGAYGFQSKKNDQQIFLFRPDSDYVAGYHVDAKSDAGWVNDKLDRRLSEISEFCSKATQP

157 ATFILP**FVEMPTD**ITKGVQHQVLLTISYDPKSKQLTPTVYDSIGRDTYSESLSSYFKGKYRTTCDEILTQSIEKAIKS
α3 helix Catalytic domain

235 TDFTLGKFTRAAYNHQNRLTEGNCGSYTFRTIKEVISSSAQGTEVKIPGSGYITSNSYLTSQHVQDIESCIKYRNLGV

313 VDIESALTEGKTLPVQLSEFIVALEDYGKLRSQQSEKSMLNFIGYSKTAKLTAVELLIGILNDIKGKNEISESQYDKL
MT domain

391 VKEVDCLMDSSLGKLVQFHLKNLGAESLQKLVLPCVKFDDTIDDFVTIEKDELFDVPDITGEELASKKGIEQGALDKE
LIR3

469 ALLKQKQIKTDLLDLREEDKTGLKKPLHGGIKVK.

B

IVDE**FEEL**GEQESDIDE**FDLL**EGD RavZ LIR1/2
CVKFDDTIDD**FVTI**EKDELFDVPD RavZ LIR3
TDYRPPDDAV**FDII**TDEELCQIQE Fyco1
SKNSSCDTDD**FVLV**PHNISSDHSC ULK2

Supplemental Figure 1. **Amino acids sequences of RavZ and LIR motifs.** (A) Amino acids sequence of RavZ protein. Core residues of LIR motifs are underlined. A catalytic domain is indicated by the thick line. Membrane-targeting (MT) domain is indicted by the dashed line. α3 helix domain is bolded. (B) Alignment of LIR motifs from RavZ, Fyco1,or ULK2. Core LIR motifs are bolded and underlined. Acidic amino acids (D/E) are colored in red.

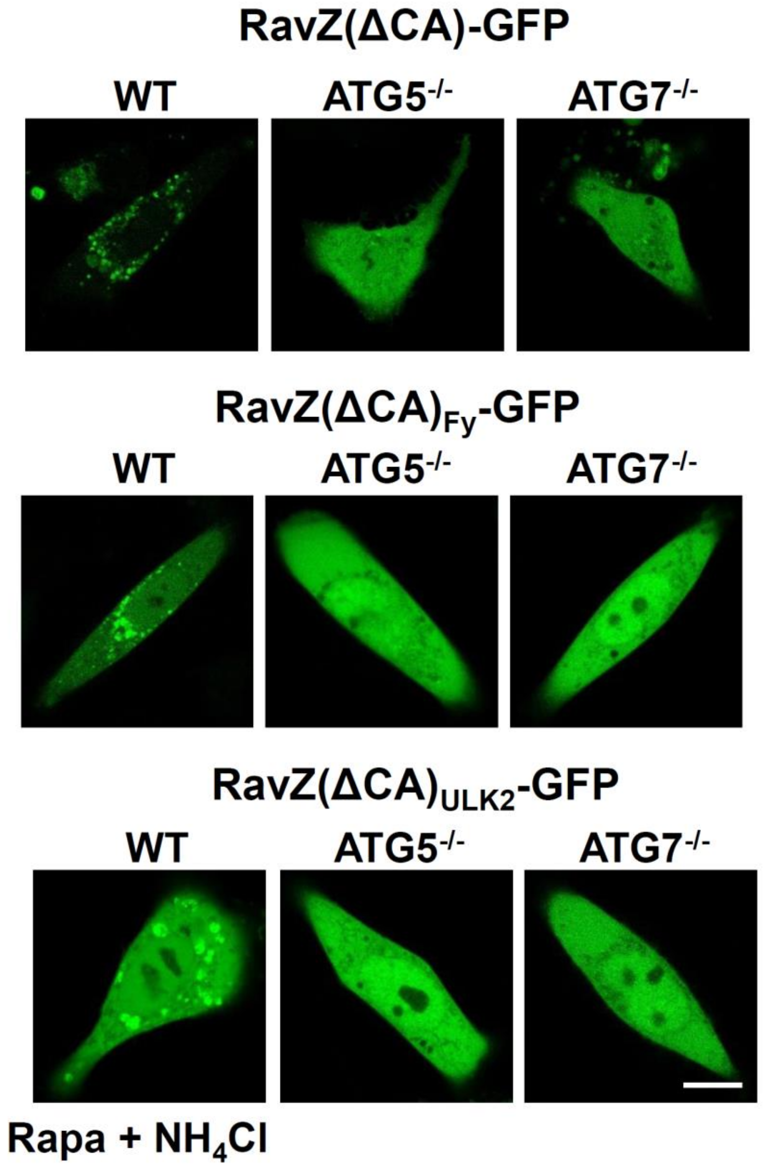

Supplemental Figure 2. Cellular localization of RavZ(ΔCA)$_X$-GFP in autophagy-deficient HeLa cells. (A) Cellular localization of RavZ(ΔCA)-GFP, RavZ(ΔCA)$_{Fy}$-GFP or RavZ(ΔCA)$_{ULK2}$-GFP in vehicle or rapamycin (rapa)/NH_4Cl-treated wild-type (WT), ATG5-knockout (ATG5$^{-/-}$), or ATG7-knockout (ATG7$^{-/-}$) HeLa cells. Scale bar, 10 µm.

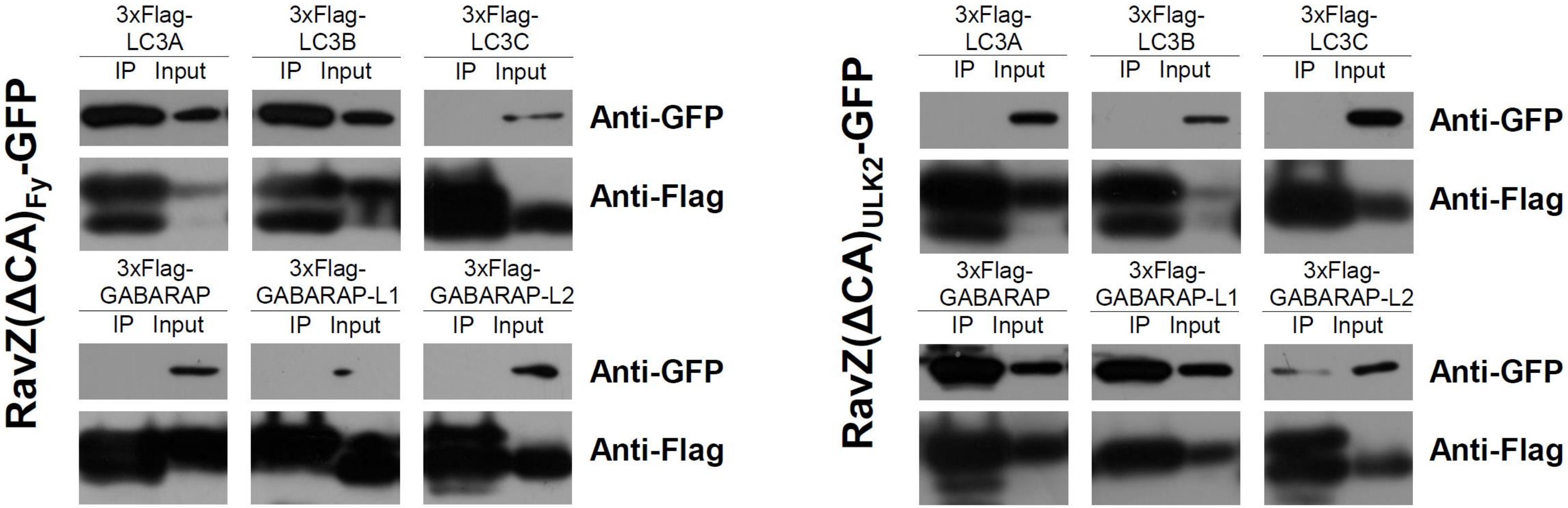

Supplemental Figure 3. Binding of mATG8 to RavZ(ΔCA)$_X$-GFP. Selective LC3A/B or GABARAP subfamily binding properties of RavZ(ΔCA)$_{Fy}$-GFP or RavZ(ΔCA)$_{ULK2}$-GFP, respectively. The cells extract was immunoprecipitated with anti-Flag antibody.

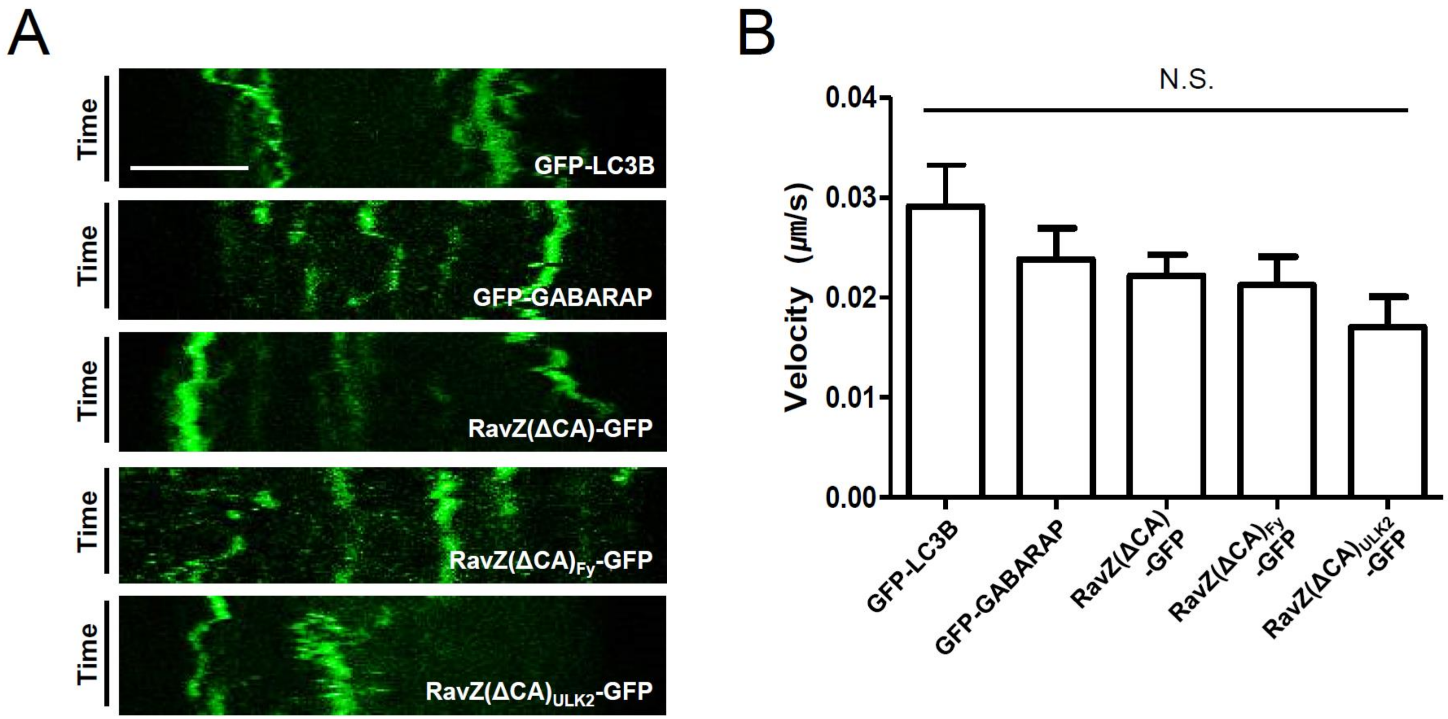

Supplemental Figure 4. Effects of GFP-mATG8 or RavZ(△CA)$_X$-GFP on autophagosome movement upon autophagy induction. (A) Kymographs showing the movement of autophagosomes with expression of GFP-LC3, GFP-GABARAP (GFP-RAP), RavZ(△CA)-GFP, RavZ(△CA)$_{Fy}$-GFP or RavZ(△CA)$_{ULK2}$-GFP in MEFs upon rapamycin (100 nM for 4 hrs) treatment for 5 min. Scale bar, 10 μm. (B) The bar graph indicates the velocity of autophagosome cell (*n* =30 for each group). Bar graph is presented as the mean ± SEM. P = 0.137, One-way ANOVA. N.S., not significant.

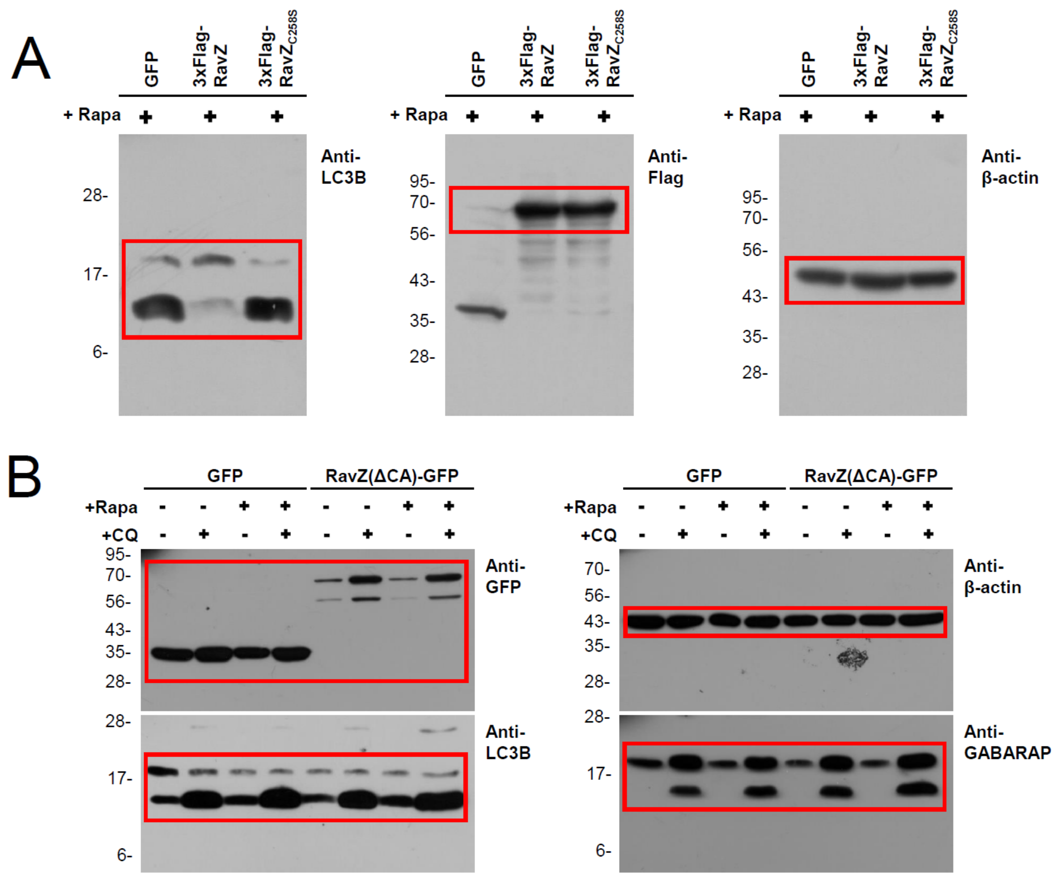

Supplemental Figure 5 (Figure 1A and F in the main text). Full blot images including the data presented in Figure 1A (A) and Figure 1F (B). Red box indicates the cropped gel blot presented in Figure 1A and 1F.

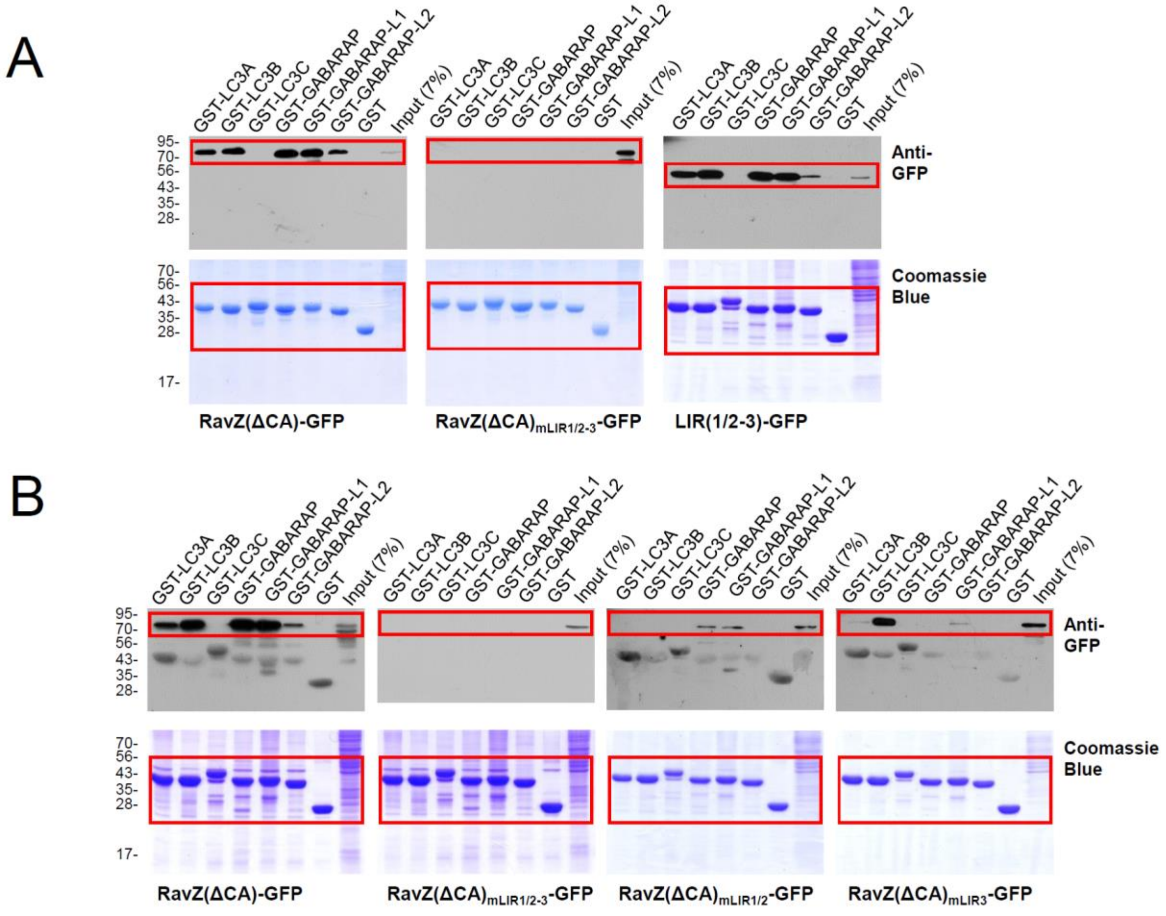

Supplemental Figure 6 (Figure 2C and Figure 3C in the main text). Full blot images including the data presented in Figure 2C (A) and Figure 3C (B). Red box indicates the cropped gel blot presented in Figure 2C and 3C.

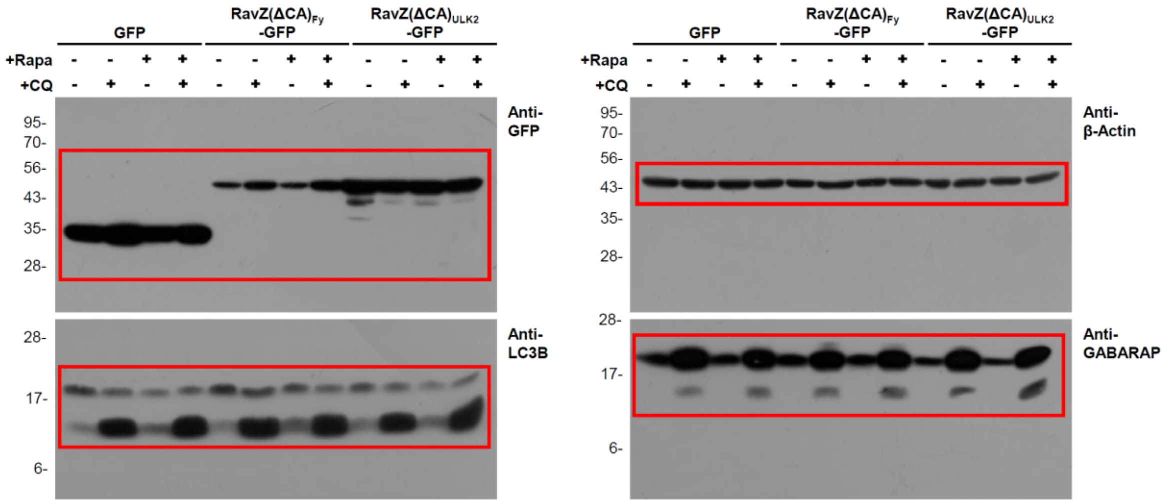

Supplemental Figure 7 (Figure 5E in the main text). Full blot images including the data presented in Figure 5E Red box indicates the cropped gel blot presented in Figure 5E.

Supplemental Table 1. Summary of the A/C ratio and mATG8 binding property of the constructs used in the experiments.

A/C ratio: 0-2.0: ; 2.0-4.0: ; 4.0-6.0: ; 10.0-12.0: ; 12.0-:

Probe	A/C ratio in each ATG8 expressing cells						mATG8 binding (From GST pulldown assay)
	LC3A	LC3B	LC3C	GABARAP	L1	L2	
RavZ(ΔCA)-GFP	-	11.6 (±0.24; N=150)	-	11.5 (±0.22; N=150)	-	-	LC3A/B, GABARAP/-L1/L2
LIR1/2-3-GFP	-	2.00 (±0.05; N=75)	-	2.02 (±0.06; N=75)	-	-	LC3A/B, GABARAP/-L1/L2
RavZ(ΔCA)$_{mLIR1/2-3}$-GFP	-	1.11 (±0.02; N=150)	-	1.07 (±0.01; N=150)	-	-	None
RavZ(ΔCA)$_{mLIR1/2}$-GFP	-	4.05 (±0.13; N=75)	-	4.78 (±0.11; N=75)	-	-	GABARAP/-L1
RavZ(ΔCA)$_{mLIR3}$-GFP	-	3.29 (±0.15; N=75)	-	1.35 (±0.04; N=75)	-	-	LC3B and weak to GABARAP-L1
GFP-LIR3	-	1.01 (±0.02; N=75)	-	1.03 (±0.02; N=75)	-	-	N.D.
GFP-MT-LIR3	-	5.21 (±0.20; N=75)	-	5.10 (±0.201; N=75)	-	-	N.D.
HyD-GFP-LIR3	-	2.29 (±0.08; N=75)	-	2.70 (±0.08; N=75)	-	-	N.D.
RavZ(ΔCA)$_{Fy}$-GFP	14.5 (±0.20; N=75)	13.5 (±0.19; N=75)	4.02 (±0.15; N=75)	2.37 (±0.12; N=75)	2.70 (±0.13; N=75)	2.80 (±0.12; N=75)	LC3A/B and weak to LC3C
RavZ(ΔCA)$_{ULK2}$-GFP	2.787 (±0.11; N=75)	2.71 (±0.10; N=75)	5.07 (±0.17; N=75)	14.5 (±0.29; N=75)	12.5 (±0.29; N=75)	11.7 (±0.22; N=75)	GABARAP/-L1/L2

* N.D.: Not determined

Supplemental Table 2. Primer sequences used for the experiments.

Construct	Primer sequences (5'-3')
LIR1/2 motifs	Forward: CGCCCAAGCTTGCCACCATGATAGTGGATGAGTTTGAA<br>Reverse: GACGGTACCCTCATCACCTTCAAGAAG
LIR3 motif	Forward: CGACCGCTCGAGACGATAGATGATGCTGTG<br>Reverse: ATAAGAATGCGGCCGCCTATTTTACCTTAATGCCACC
Membrane-targeting (MT) domain	Forward: CTAGGGCTAGCGCCACCATGCCGGTACAGCTTTCTGAA<br>Reverse: GACGGTACCATCATCAAACTTGACACA
HyD domain	Forward: GGAAGAAGAACAACACGAGCCCCTGCCCGCC<br>Reverse: CAGGGGCTCGTGTTGTTCTTCTTCCAT
LIR1 mutant	Forward: GAGGCTGAAGAAGCGGGAGAACAGGAATCCGAT<br>Reverse: TCCCGCTTCTTCAGCCTCATCCACTATTAATTT
LIR2 mutant	Forward: GAAGCTGATCTTGCTGAAGGTGATGAGAAATTG<br>Reverse: TTCAGCAAGATCAGCTTCATCGATATCGGATTC
LIR3 mutant	Forward: GATGCTGTGACTGCCGAAAAAGACGAACTATTC<br>Reverse: TTCGGCAGTCACAGCATCATCTATCGTATCATC
LIR motif from Fyco1	Forward: CCCAAGCTTGCCACCATGACAGACTACAGGCCACCGGACGACGCT<br>Reverse: GACGGTACCGGACTCCTGTATCTGGCA<br>Forward: GCAGAATTCAGGCCACCGGACGACGCT<br>Reverse: ATAAGAATGCGGCCGCCTAGGAGCCGGACTCCTGTAT
LIR motif from ULK2	Forward: CGCCCAAGCTTGCCACCATGAGCAAGAACTCTTCTTGT<br>Reverse: GACGGTACCATCACATGAGTGGTCTGACGAGATGTTGTGTGGCA<br>Forward: GCAGAATTCAGCAAGAACTCTTCTTGT<br>Reverse: ATAAGAATGCGGCCGCCTAATCACATGAGTGGTCTGA
